# Chromium Doped UO_2_-Based Ceramics: Synthesis and Characterization of Model Materials for Modern Nuclear Fuels

**DOI:** 10.3390/ma14206160

**Published:** 2021-10-17

**Authors:** Philip Kegler, Martina Klinkenberg, Andrey Bukaemskiy, Gabriel L. Murphy, Guido Deissmann, Felix Brandt, Dirk Bosbach

**Affiliations:** Forschungszentrum Jülich GmbH, Institute of Energy and Climate Research—Nuclear Waste Management and Reactor Safety (IEK-6), 52428 Jülich, Germany; m.klinkenberg@fz-juelich.de (M.K.); a.bukaemskiy@fz-juelich.de (A.B.); g.murphy@fz-juelich.de (G.L.M.); g.deissmann@fz-juelich.de (G.D.); f.brandt@fz-juelich.de (F.B.); d.bosbach@fz-juelich.de (D.B.)

**Keywords:** UO_2_, doping, wet chemical preparation, chromium, spent nuclear fuel, ceramics, model systems

## Abstract

Cr-doped UO_2_ as a modern nuclear fuel type has been demonstrated to increase the in-reactor fuel performance compared to conventional nuclear fuels. Little is known about the long-term stability of spent Cr-doped UO_2_ nuclear fuels in a deep geological disposal facility. The investigation of suitable model materials in a step wise bottom-up approach can provide insights into the corrosion behavior of spent Cr-doped nuclear fuels. Here, we present new wet chemical approaches providing the basis for such model systems, namely co-precipitation and wet coating. Both were successfully tested and optimized, based on detailed analyses of all synthesis steps and parameters: Cr-doping method, thermal treatment, reduction of U_3_O_8_ to UO_2_, green body production, and pellet sintering. Both methods enable the production of suitable model systems with a similar microstructure and density as a reference sample from AREVA. In comparison with results from the classical powder route, similar trends upon grain size and lattice parameter were determined. The results of this investigation highlight the significance of subtly different synthesis routes on the properties of Cr-doped UO_2_ ceramics. They enable a reproducible tailor-made well-defined microstructure, a homogeneous doping, for example, with lanthanides or alpha sources, the introduction of metallic particles, and a dust-free preparation.

## 1. Introduction

The introduction of modern nuclear fuels such as those including Cr-, Al-, and Si- doped types [1,2,3] has led to a significant improvement in the performance of conventional light water reactors (LWR) over traditional non-doped forms. Specifically, modern fuels provide a reduction in fuel swelling and amelioration in heat transfer, leading to higher burn up and power output [4,5,6,7]. The physical origin behind these enhanced properties, in the case of the Cr-doped fuels, is due to the induced coarse-grained microstructure with a narrow grain size distribution. This increases the retention of gaseous fission products inside the fuel during operation due to improved diffusion behavior compared to conventional LWR fuels [8]. During the production of Cr-doped fuels, the UO_2_ matrix can only incorporate Cr in trace amounts of up to approximately 1500 ppm [9,10]. Concurrently, some of the Cr precipitates as a separate phase typically located at the grain boundaries of the UO_2_ matrix [8,11]. The introduction of modern, Cr-doped, nuclear fuels at present, is still a relatively recent addition to current reactor fleets. Subsequently, key questions remain regarding their properties and behavior, making them a topical focus of recent fuel investigations.

Similar to conventional UO_2_-based LWR fuels, spent modern fuels are likely to be disposed of in deep geological repositories, which are considered the most appropriate disposal option for high level nuclear waste [12,13,14]. However, knowledge gaps still exist on the stability of the spent modern fuels at conditions of a deep geological waste repository. Spent modern fuels, like all UO_2_ based spent fuels, are complex multi-phase ceramics that are associated with high beta- and gamma-radiation fields. This makes unravelling the various concurring dissolution mechanisms directly from experiments with spent modern fuels challenging.

Model systems like SIMFUEL were first developed to mimic all aspects of certain spent nuclear fuel types except for the radiation fields [15,16]. However, it became apparent that the sheer complexity of SNF varieties could not be simplistically captured in multi-component systems like SIMFUEL. This led to the utilization of specific-component model system studies that focused on single effects tailored to conditions relevant to a certain SNF type/variety [17,18,19,20]. When combined systematically, such studies allow for a greater in-depth understanding that can create a near holistic viewpoint of a specific SNF type under certain relevant conditions. Critical to such an approach is the development of accurate model systems that use reliable and replicable methods. These are well established for traditional UO_2_ fuels, but less so for modern fuels such as Cr doped UO_2_. Recent studies [9,21,22] on Cr doped UO_2_ have typically utilized powder-milling methods involving UO_2_ and Cr_2_O_3_ powders. Although these are more time-efficient in execution than methods such as sol-gel or co-precipitation, they often result in less homogeneous samples. In terms of data interpretation and analysis, this is a critical issue since the addition of ppm levels of Cr will result in subtle measurable effects occurring that will not be representative under inhomogeneous conditions. Accordingly, to follow a systematic approach in the investigation of modern fuels from simple to multi-component systems, consistent and more efficient preparation routes need to be developed, other than that of powder mixing, for example [23]. By design, such methods should be transferable to other homogeneous additions in UO_2_ ceramics, for instance, Pu or lanthanides in the context of MOX and SNF studies, respectively.

Interest in the incorporation of Cr into UO_2_ has recently expanded from that of applied SNF related to a more general inorganic structural-chemical related, as the mechanism for incorporation has been called into question by Sun et al. (2020) [24]. It has been typically presumed that the use of Cr_2_O_3_ coupled with its stable electronic configuration ([Ar]3*d*^3^) results in the trivalent state solely entering the ceramic, akin to the behavior of *Ln*^3+^ in UO_2_. However, a recent investigation by [24] using ab initio simulations and re-evaluated XANES measurements by Mieszczynski et al. (2014) [25] argued that instead of Cr^3+^ within the UO_2_ fuel matrix, Cr exists as Cr^2+^ with U^5+^ and an oxygen vacancy surrounding it. Further calculations by Cooper et al. (2018) argue that under high temperatures, Cr can exist monovalently with excess oxygen vacancies facilitating this [26]. Several investigations have attempted to resolve the issue through X-ray absorption spectroscopy (XAS) on bulk samples. However, the low solubility of Cr results in variable amounts of Cr_2_O_3_ precipitates forming along the grain boundary intersections. This results in convoluted measurements of which true observation of Cr inclusion in the UO_2_ matrix and not grain boundary and precipitate regions becomes difficult. Although [25] argued that Cr^2+^ is observable, it is not clear whether it is from the matrix and/or the grain boundary/Cr precipitate regions. Notably and relevant to the present investigation, is that the aforementioned experimental studies have typically relied upon powder-milling produced Cr doped UO_2_ samples. Nevertheless, the chemical state of Cr within the UO_2_ lattice experimentally can still be considered an open question and for it to be answered, appropriate and representative model systems are required. Therefore, the usage of the term “Cr_2_O_3_” for structurally incorporated chromium within this work is only for the purpose of consistency with the previous literature.

The transformation of ammonium di-uranate ((NH_4_)_2_U_2_O_7_, ADU) to U_3_O_8_ and finally to UO_2_ is a well-established wet chemical route leading to good sinterability of UO_2_ pellets. In addition, earlier studies by Hung et al. (2001) [27] showed that the route can be optimized to avoid lubricants (e.g., Zn-stearate) or sintering aids during compaction and sintering, providing the potential for single effect studies, even when only trace amounts of additional elements are used. Here, we present two new, wet chemical approaches for the preparation of Cr-doped UO_2_ based model systems for single effect dissolution studies related to modern fuels. A parameter variation study was carried out including optimization of powder synthesis, green body compaction, and variation of the oxygen potential. Detailed investigations were carried out on the effects of the individual parameters on the microstructure, density, grain growth of the ceramics, and the structural uptake of Cr into UO_2_ as well as the formation of separate Cr-rich precipitates. The effect of adding chromium nitrate at different stages of the synthesis route to UO_2_ was tested and compared to results of the common powder syntheses. Finally, the results were compared to pure UO_2_ pellets, produced within the framework of this work, used as reference for the single effect studies and to an industrially produced, chromium doped UO_2_ pellet made by AREVA used as the reference material for fresh nuclear fuel.

## 2. Materials and Methods

### 2.1. Preparation of a Cr-Doped UO_2_ Based Ceramic

The preparation was adapted from the general procedure of [28] for the wet chemical preparation of ammonium di-uranate ((NH_4_)_2_U_2_O_7_, ADU) from uranyl nitrate solution. The main steps: (1) powder preparation; (2) Cr-doping; and (3) preparation of ceramic pellets and their optimization, are described in detail below. Two different methods for the chromium doping of the pellets were applied: (1) a co-precipitation method (CPM) and (2) a wet coating method (WCM) as adapted from [29,30,31].

#### 2.1.1. Preparation of Pure UO_2_-Powder 

The precursor powders for all pellets presented in this work were produced by precipitation of ADU from an aqueous solution containing 2 M uranyl nitrate (UO_2_(NO_3_)_2_, Merck, p.a.) using a 16.5 M ammonia solution (Merck). The reaction was carried out with an excess of ammonia of at least 300% to adjust the equilibrium to the side of the products, according to:

2 UO_2_(NO_3_)_2_ + 6 NH_3_ + 3 H_2_O → (NH_4_)_2_U_2_O_7_ + 4 (NH_4_)NO_3_ (1)

The uranyl nitrate solution was stepwise pipetted into the ammonia solution, which was constantly mixed by a magnetic stirrer. After complete addition of the solution, the mixture was stirred for 2 h. This led to the formation of ADU as a yellow precipitate, which was then separated from the solution by centrifuging for 5 min and afterward decanting the solution. The precipitate was then washed three times with high purity water to remove excess (NH_4_)NO_3_ and NH_3_. During the final washing step, water was replaced with ethanol and the powder was left to dry. ICP-MS measurements of the supernatant solution showed that about 99% of the uranium from the stock solution was precipitated as ADU.

The dried ADU powder was calcined under air for denitrification, dehydration, and transformation to U_3_O_8_ using a box furnace (Carbolite CWF 13/5, Neuhausen, Germany). In order to optimize the influence of the temperature on the decomposition, a series of ADU-powders was prepared and calcined at temperatures of 450, 600, 700, 800, and 900 °C, respectively, for 5 h. The uncertainties of the temperature determination for this step of thermal treatment are ±5° C, based on the manufacturer’s information.

The resulting U_3_O_8_ powder was reduced to stoichiometric UO_2_ via a 5 h thermal treatment in a tube furnace (ENTECH ESTF 50-18-SP-VK, Ängelholm, Sweden) under a 4% H_2_—96% Ar mixture (HYTEC). The completeness of the reduction as well as the effect of temperature on the final density of the sintered pellets was investigated by performing the reduction at three different temperatures, namely 600, 800, and 900 °C. Uncertainties for the temperature determination of both steps are ±5° C each, based on the manufacturer’s information.

#### 2.1.2. Cr Doping

Depending on the doping method, chromium was added at different stages of the ceramic’s preparation. For the powders produced via the CPM approach, the desired amount of chromium was added to the uranyl nitrate solution as 0.02 M chromium(III) nitrate nonahydrate solution (EMSURE^®^, M-Clarity™ quality level = MQ300). During the precipitation of ADU (Equation (1)), chromium is quantitatively precipitated as Cr(OH)_3_ according to

Cr(NO_3_)_3_ + 3 NH_3_ + 3 H_2_O → 3 (NH_4_)NO_3_ + Cr(OH)_3_ (2)

Materials with doping levels of 1000 and 2500 ppm Cr_2_O_3_ were precipitated and thermally treated in the same way as the pure ADU at 600 °C for calcination and reduction, respectively.

For the WCM preparation, a pure UO_2_ from the wet chemical route according to Section 2.1.1. was used after a complete thermal treatment at 600 °C for both steps. The UO_2_ was wet coated with a chromium nitrate nonahydrate solution (0.02 M) in appropriate amounts corresponding to initial doping levels of 1000, 1500, and 2500 ppm Cr_2_O_3_. The uncertainty of the initial doping level was derived from the errors of the balance and pipetting devices as below 2%. 

In order to distribute the Cr homogeneously on the grain surfaces, suprapure water was admixed until the complete powder was covered with liquid. After intensive stirring, this mixture was dried and subjected to the same thermal treatment as the original pure UO_2_. Earlier studies showed that about 20 to 40% of the chromium initially added was lost to the furnace atmosphere during sintering, so all data described here are related to the initial doping level [9]. 

#### 2.1.3. Preparation of Ceramic Pellets

In order to obtain disk shaped green bodies 10 mm diameter, about 1 g of the calcined powders were compacted for 10 s with a tungsten carbide piston of an uniaxial press (Hahn & Kolb, MP12, Stuttgart, Germany), without any addition of a binder or lubricant. The optimum preparation of the green body was tested according to an approach of Bukaemskiy et al. (2009) and Babelot et al. (2017) [32,33], who in detail tested the compressibility and sinterability of oxide ceramic powders synthesized by different wet chemical methods. Within a log unit (compaction) pressure versus relative green density plot, they identified three linear regions, the low, intermediate, and high pressure region and an optimal pressure corresponding to the highest sintered density, located in the upper part of the intermediate-pressure region. In the present work, the compressibility and sinterability of the studied powders were tested in the compaction pressure region from 190 to 760 MPa to identify the optimum compaction pressure. Uncertainty of the pressure during compaction was estimated due to the setup of the press to be 0.1 MPa. After pressure release, the green body was carefully pressed out of the die-plate using the lower piston of the uniaxial press and placed in a corundum crucible for subsequent sintering. The density of an ideal UO_2_ crystal as directly calculated from lattice parameters (10.958 g/cm^3^), the mass of UO_2_ of the green body, and its height were used for the calculation of the green density [34]. Due to the low doping levels, all green densities and sintered densities refer to an ideal pure UO_2_ crystal.

For the sintering of the pellets, the crucibles with the green bodies were placed in the hot zone of a horizontal tube furnace (ENTECH ESTF 50-18-SP-VK, Ängelholm, Sweden). The pellets were sintered at 1700 ± 5 °C for 10 h. Heating rates were 4 °C/min, and cooling rates were 6 °C/min. During the sintering process, the furnace was flushed with 1600 mL/min of a 4% H_2_–96% Ar mixture, or with the less reducing mixture resulting from mixing 1567 mL/min of 4% H_2_–96% Ar and 33 mL/min of 1% H_2_–99% Ar. This leads to two different values of oxygen partial pressure during the sintering process. The required proportions of the gas mixtures were calculated beforehand to achieve the desired oxygen partial pressures. In addition, the oxygen partial pressure was monitored during the sintering process using a yttrium stabilized ZrO_2_ oxygen sensor.

Oxygen partial pressure (pO_2_) can also be expressed as relative partial molar Gibbs free energy of oxygen, or oxygen potential, P_O2_:

ΔG° = RT ln P_O2_ (3)

where G° is the standard free energy of O_2_; P_O2_ is the (pO_2_/p°); and p° is the standard pressure.

The oxygen potential for the 4% H_2_–96% Ar mixture was −510 kJ/mol O_2_, the less reducing mixture was prepared by mixing certain amounts of 4% H_2_–96% Ar with 1% H_2_–99% Ar to achieve an oxygen potential of −420 kJ/mol O_2_. As high purity gases were used, the uncertainty of the oxygen potential was less than 1%.

### 2.2. Characterization

#### 2.2.1. Physical Properties

The densities of the pellets were determined by the geometrical method for the green bodies. The density of the sintered pellets and the closed porosity were measured by the hydrostatic weighing method in water. The open porosity was estimated via a penetration-immersion method [35,36] with hot paraffin (150 °C) as the impregnation fluid. The measurements were performed with a Mettler Toledo density determination kit in combination with a Mettler Toledo AT261 (Giessen, Germany) precision balance. The theoretical densities of the materials (ρT) were calculated from the results of the X-ray diffraction (XRD) measurements as described in [37]. All densities described later are relative densities, provided in percent of the theoretical density. Uncertainties of all determined densities are 2 sigma deviations and are provided in Table S1.

#### 2.2.2. Crystal Structure

XRD analyses were performed with a D4 Endeavor diffractometer in Bragg–Brentano geometry (Bruker AXS GmbH, Karlsruhe, Germany) using Cu radiation (λ CuK_α1_ = 1.5405 Å) at a power setting of 40 kV and 40 mA. A linear silicon strip LynxEye detector (Bruker-AXS, Karlsruhe, Germany) was used to determine the crystal structure of the powder and pellet samples. The aperture of the motorized divergence slit was set to 0.3 mm and the receiving slit to 8.0 mm, respectively. XRD patterns were collected from the final pellets at ambient conditions in the 2Θ range from 10 to 120° using a step size of 0.01°/2Θ and a counting time of 2 s per step.

The structure of UO_2_ in different pellets was refined by the Rietveld method as implemented in the GSAS2 software [38,39]. The peak shapes were modeled using a pseudo-Voigt function and the background was estimated using a 12–18 term shifted Chebyschev function. The scale factor, detector zero-point, and lattice parameters were refined together with the peak profile parameters.

#### 2.2.3. Microstructure

Surfaces of the sintered pellets were carefully polished with a Struers Rotopol 22/Struers Roto Force 4 polishing device (Ballerup, Denmark) using diamond pastes (up to 1 µm) and finally a colloidal Si-suspension (OP-S, 0.25 µm, Struers, Ballerup, Denmark) for further investigations of the pellets’ microstructure.

Microstructural observations on powders and ceramic pellets were carried out with an environmental scanning electron microscope (SEM, Quanta 200F, FEI, Eindhoven, The Netherlands) in low vacuum mode at a pressure of 60 Pa. Energy-dispersive X-ray spectroscopy (EDS) employing an Apollo X detector system (EDAX, Weiterstadt, Germany) was used to determine the chemical composition of the synthesized solids and to assess their chemical homogeneity. EDS spot analyses as well as EDS elemental mappings, to determine the spatial distribution of Cr, were carried out at 20 kV and a working distance of 10 mm. Image analyses of EDS mappings were applied using the software package Fiji (ImageJ, Bethesda, USA). To assess possible variations in the average grain size across the pellets, SEM images were taken along the diameter of the pellets in three fields: edge, midway, and center. The grain size was determined using the intercept method ASTM-E112-13 as described in the ASTM standards [40]. Uncertainties of the grain sizes are 2 sigma deviations and listed in Table S1 in supplementary materials.

## 3. Results

In the following, first, the optimization of the important steps for the preparation of Cr-doped UO_2_ ceramics is addressed. In the second part of the results section, a detailed parameter study of the consequences of Cr-doping upon the physical properties, crystal structure, and microstructure of the UO_2_ based ceramics is presented.

### 3.1. Optimisation of the Synthesis Route

#### 3.1.1. Powder Preparation and Pellet Density: Pure UO_2_

Two important aspects of the powder preparation route were examined: (1) temperature, and (2) the corresponding powder reactivity during the production of the green body of the UO_2_ based ceramic including a variation of the pressure applied during green pellet preparation. 

**Figure 1:**
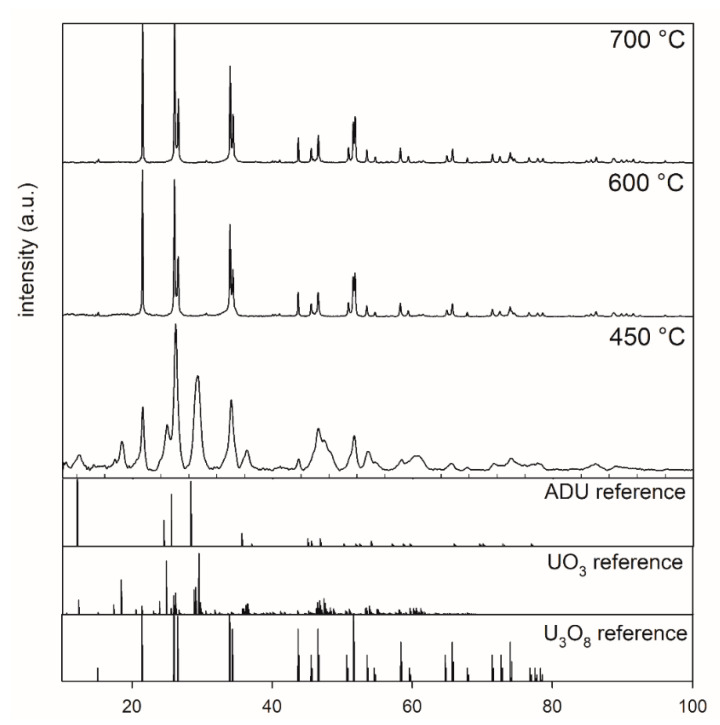
XRD patterns of powders after calcination at 450 °C, 600 °C, and 700 °C collected between 10° and 100° 2 theta, highlighting the effect of calcination of ADU (5 h, air) at different temperatures.

Figure 1 shows the XRD patterns of ADU powders after thermal treatment at 450, 600, and 700 °C for 5 h. Inspection of the XRD pattern for the 450 °C sample indicated that the sample did not completely transform to U_3_O_8_. Phase analysis indicated that this sample contained a mixture of U_3_O_8_, ADU, and UO_3_. The XRD patterns of powders calcined at temperatures higher than or equal to 600 °C showed a complete transformation from ADU to U_3_O_8_.

**Figure 2:**
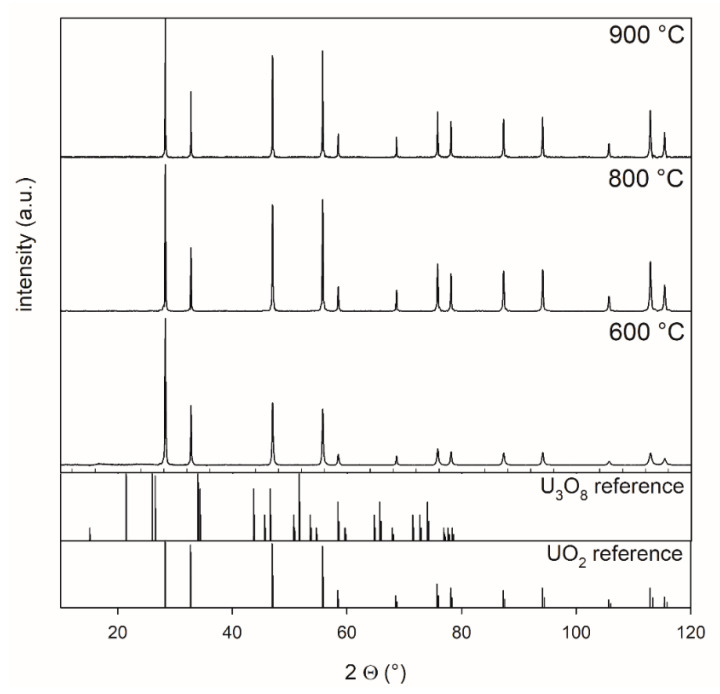
XRD patterns of powders reduced at 600 °C, 800 °C, and 900 °C collected between 10° and 120° 2 theta, highlighting the effect of the reduction of U_3_O_8_ for 5 h in a 4% H_2_–96 % Ar mixture at different temperatures. Oxygen potential is −510 kJ/mol O_2_.

The XRD patterns of the products after the second, reducing, thermal treatment are shown in Figure 2. U_3_O_8_ powder, which was synthesized from ADU at 800 °C, was treated at 600, 800, and 900 °C for 5 h in a 4% H_2_–96% Ar mixture. The measurements showed that a complete transformation from U_3_O_8_ to UO_2_ occurred at all temperatures.

**Figure 3:**
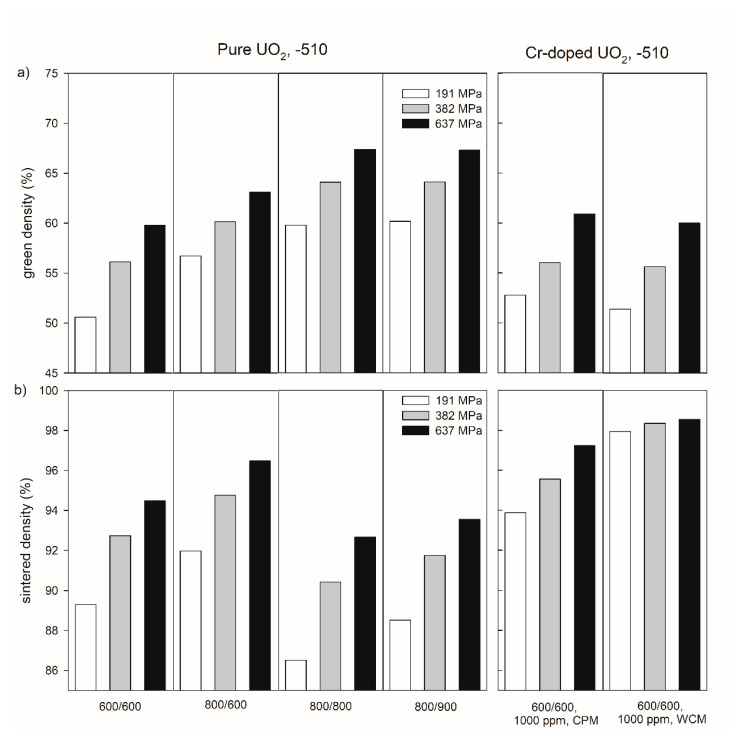
Green density (**a**) and sintered density of pure and Cr-doped UO_2_ pellets (**b**) produced at compaction pressures of 191, 382, and 637 MPa with different thermal history of the precursor materials. The numbers under the samples refer to the temperatures (°C) during the oxidative and reducing steps of the thermal treatment of the precursors. CPM = co-precipitation method, WCM = wet coating method. All pellets were sintered at an oxygen potential of −510 kJ/mol O_2_.

Figure 3 exemplarily shows the influence of temperature during the thermal treatment of the precursor materials on the density of the green bodies and pellets sintered at −510 kJ/mol O_2_ for both pure and Cr-doped materials for three compaction pressures (191, 382, and 637 MPa). In the pressure range investigated in this work, a compaction pressure increase led to an increasing green and sintered density (see also Table S1 in the supplementary information). Reducing the temperature of the first thermal treatment from 800 °C to 600 °C led to a significant decrease in green density. For example, at a compacting pressure of 637 MPa, the values of the green densities were 63.1% for 800 °C and 59.8% for 600 °C, respectively (Figure 3a, Table S1 in supplementary materials). After the subsequent sintering, the sintered density was lower for the sample with the lower green density (Figure 3b). Increasing the second step of thermal treatment from 600 °C to 800 °C led to an increase in the green density (Figure 3a), but to a decrease in the sintered density (Figure 3b). 

Chromium doping led to green densities similar to those of pure UO_2_ treated at the same temperatures (600 °C/600 °C, Figure 3 a), but to much higher sintered densities (e.g., 97.24% for CPM and 98.55% for WCM at 637 MPa, Figure 3 b). Additionally, WCM Cr-doping results in a less pronounced pressure dependence compared to pure UO_2_ and material doped by CPM. 

**Figure 4:**
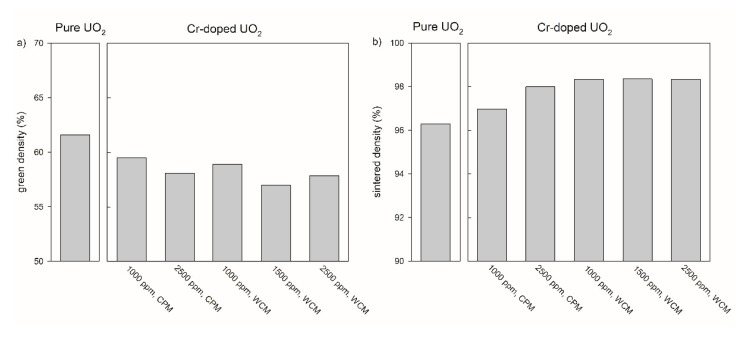
Green density (**a**) and sintered density (**b**) of pure and chromium doped UO_2_ pellets produced at a compaction pressure of 637 MPa with different doping methods and doping levels. The numbers under the sample refer to the (nominal) amount of Cr-doping. CPM = co-precipitation method, WCM = wet coating method. All pellets were sintered at an oxygen potential of −420 kJ/mol O_2_.

In addition to the sintering temperature and the compaction pressure, the sintered density of pure UO_2_ pellets depends on the oxygen potential in the sintering atmosphere (Figure 4). In case of the pure UO_2_ samples after the pre-treatment at 600 °C for both calcination steps, an increasing sintered density was observed with increasing oxygen potential from 94.5 % at −510 kJ/mol O_2_ to 96.3% at −420 kJ/mol O_2_. 

#### 3.1.2. Adaption of Green Body Preparation and Pellet Density for Cr_2_O_3_ Doped UO_2_

The relationship between compaction pressure and sintered density was similar for the samples doped with 1000 ppm Cr_2_O_3_ obtained by the co-precipitation method and for pure UO_2_ (Figure 3). However, the relative densities of the 1000 ppm Cr_2_O_3_-doped UO_2_ increased on average by 3% compared to pure UO_2_. An increase in the Cr_2_O_3_-doping level from 1000 to 2500 ppm raised the sintered density from 97.3 to 98.1 % at *P* = 637 MPa (Figure 4). For the samples doped with 1000 ppm Cr_2_O_3_ by CPM, the sintered densities of the pellets were slightly dependent on the compaction pressure, but densities were significantly higher than for pure UO_2_ and reached 98.6% at *P* = 637 MPa. All samples doped by WCM, independently on the doping level (1000, 1500, and 2500 ppm), showed similar sintered densities of nearly 98.4%. The sintering at different oxygen potentials (−510 or −420 kJ/mol O_2_) was found to have very little effect on the density of Cr_2_O_3_-doped pellets, independent of the doping level and the synthesis route (Figure 4). 

Figure 3b and Figure 4b demonstrate that the highest sintered densities are achievable using pre-treatment at 600 °C for both calcination steps, via the WCM approach and with an oxygen potential of −420 kJ/mol O_2_. The effect of the oxygen potential on microstructural features other than the density will be described in the following sections.

### 3.2. Cr_2_O_3_—UO_2_ Solid Solution Formation: Consequences on Lattice Parameters and Microstructure 

#### 3.2.1. Microstructure and Grain Size 

**Figure 5:**
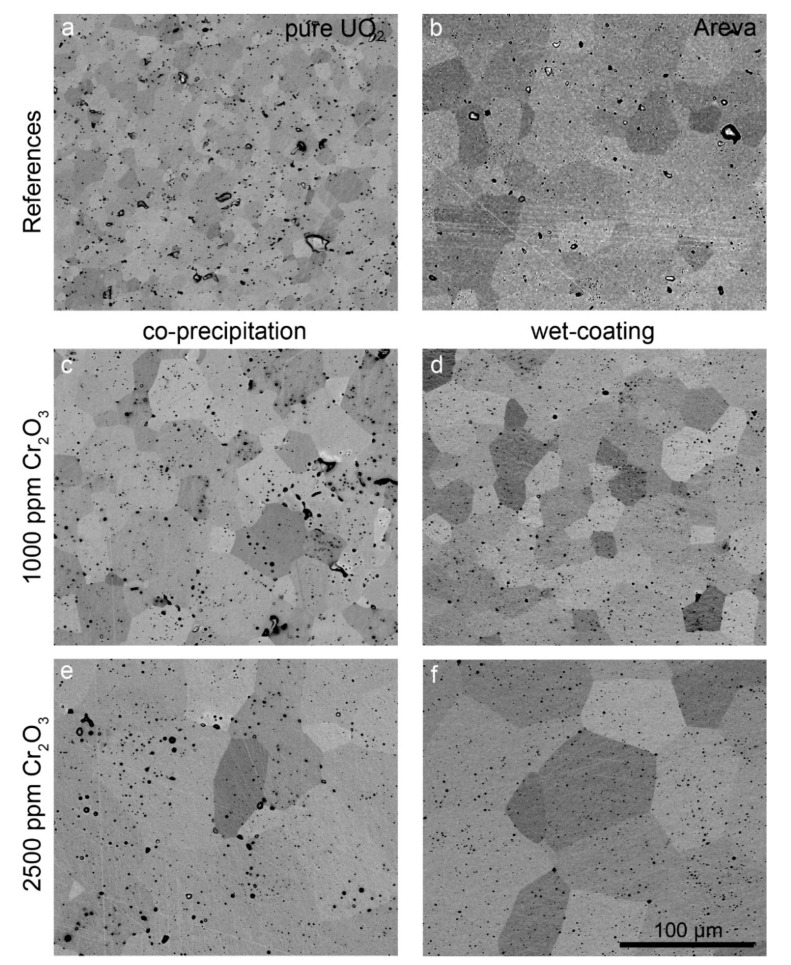
BSE images of pellets sintered at 1700 °C and an oxygen potential of −420 kJ/mol O_2_ for 10 h. (**a**) Pure UO_2_. (**b**) AREVA C_r_-doped UO_2_ sample (sintering conditions unknown). (**c**) CPM sample, 1000 ppm Cr_2_O_3_. (**d**) WCM sample, 1000 ppm Cr_2_O_3_. (**e**) CPM sample, 2500 ppm Cr_2_O_3_. (**f**) WCM sample, 2500 ppm Cr_2_O_3_. The scale is the same in all images.

The microstructure and grain size of samples sintered at 1700 °C with varying oxygen potential were analyzed in detail. The microstructures of samples prepared at an oxygen potential of −420 kJ/mol O_2_ and of the AREVA reference material are presented in the backscattered electron (BSE) SEM images in Figure 5. In this SEM mode, the individual grains can be distinguished due to their orientation contrast. Pores appear as black round features, with some of them filled with Cr, as detected by EDS (see Figure 5). 

The pure UO_2_ reference pellet as prepared by our synthesis route (Figure 5a) contained grains in a size range between 1 µm (min) and ~25 µm (max) with an average grain size of 12 µm. Typical for this sample is a uniform grain size distribution and well-formed triple points with an angle of 120°. Small pores with about a 1 µm diameter are homogeneously distributed. Additionally, some pores with a size of up to 10 µm are visible. No systematic relationship between the location of the pores and grain boundaries or triple points was observed. Pure UO_2_ pellets sintered at more reducing conditions (−510 kJ/mol O_2_) showed the same microstructure (Figure S1 in supplementary materials).

**Figure 6:**
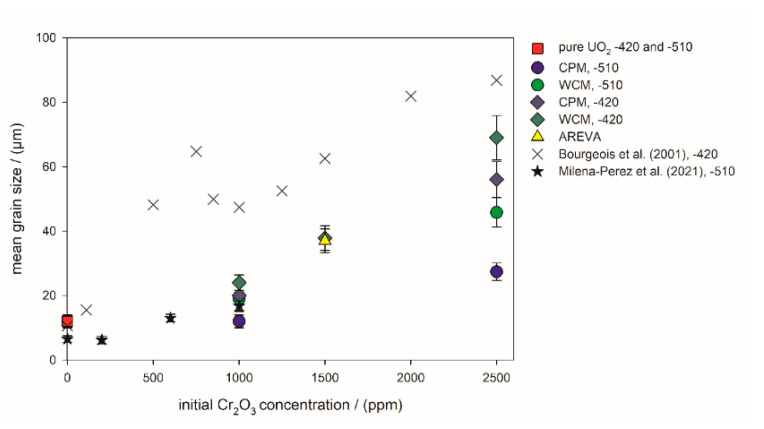
Mean grain size of the sintered pellets versus the initial Cr_2_O_3_-doping level. −510: Sintering atmosphere was Ar/4% H_2_ with an oxygen potential of −510 kJ/mol O_2_. −420: Sintering atmosphere was Ar/4% H_2_ + Ar/1% H_2_ with an oxygen potential of −420 kJ/mol O_2_. The sintering temperature of all pellets in this work was 1700 °C. Literature values were taken from Bourgeois et al. (2001) [23], Milena-Perez et al. (2021) [22] and Silva et al. (2021) [21].

In contrast to the pure UO_2_ pellet, the grains of the AREVA reference UO_2_–Cr_2_O_3_ ceramic (1500 ppm Cr_2_O_3_ as initial composition; Figure 5b) were significantly larger, with sizes in the range between 8 and 70 µm and an average grain size of 37 µm (Figure 5 and Figure 6, Table 1, Table S1 in supplementary materials). The porosity was lower than that of the pure UO_2_ reference, whereas the general microstructure concerning triple points, etc. was similar. Similarly, the WCM and CPM samples, starting from 1000 ppm Cr_2_O_3_, also had increased average grain sizes of 20 µm and of 24 µm, respectively (Figure 5c,d and Figure 6). For direct comparison with the AREVA reference, a sample with 1500 ppm Cr_2_O_3_ was prepared via WCM, which showed a similar microstructure and average grain size (Figure S2 in supplementary materials). Increasing the initial Cr_2_O_3_ doping level further to 2500 ppm led to an additional coarsening effect and average grain sizes of 56 (CPM) and 69 µm (WCM; Figure 5 and Figure 6). In general, the co-precipitation samples exhibited a slightly higher porosity and a smaller grain size than the wet coating samples. 

The mean grain sizes obtained in this study and the available literature data are compiled in Figure 6. The data from the present wet chemical syntheses show a systematic shift in the average grain size toward larger grains, resulting from the variation of oxygen potential to lower values. In general, the samples prepared at −510 kJ/mol O_2_ exhibited a lower grain size than the corresponding samples sintered at −420 kJ/mol O_2_. The grains of the WCM samples were systematically larger than the grains of the CPM samples. The AREVA pellet had an identical average grain size as the WCM sample initially doped with 1500 ppm Cr_2_O_3_ and sintered at −420 kJ/mol O_2_. Compared to the pure UO_2_ sample, the addition of 1000 ppm Cr_2_O_3_ only had minor effects on the average grain size for the samples sintered at −510 kJ/mol O_2_, and a more pronounced effect at −420 kJ/mol O_2_ (Figure 6).

**Figure 7:**
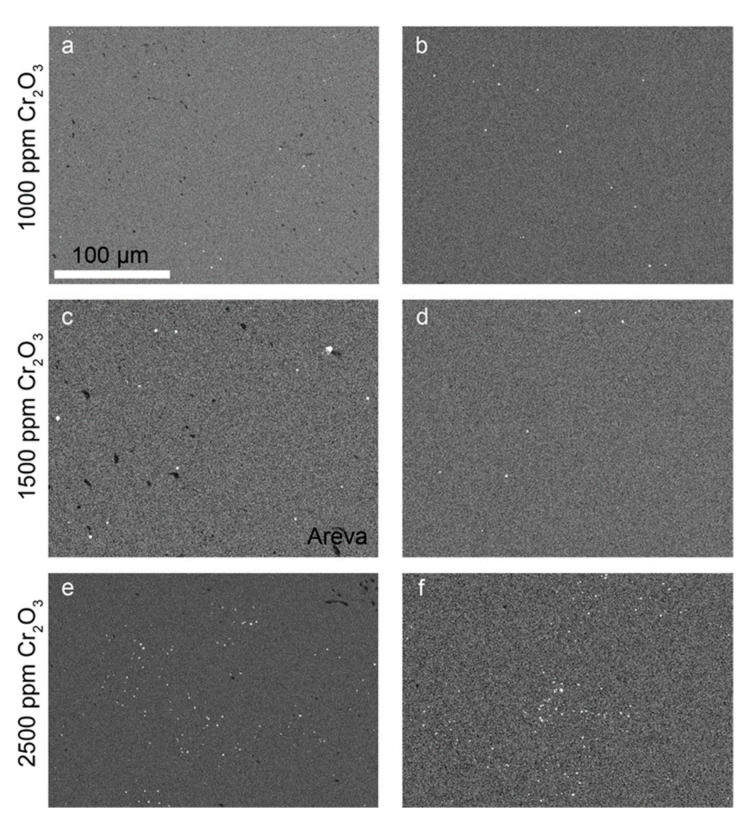
EDS-mapping of Cr–K in Cr_2_O_3_-doped samples produced by (**a**) co-precipitation with 1000 ppm Cr_2_O_3_; (**b**) WCM with 1000 ppm Cr_2_O_3_; (**c**) AREVA with 1500 ppm Cr_2_O_3_; (**d**) wet coating with 1500 ppm Cr_2_O_3_; (**e**) co-precipitation with 2500 ppm Cr_2_O_3_; and (**f**) WCM with 2500 ppm Cr_2_O_3_. All samples were sintered at an oxygen potential of −420 kJ/mol O_2_. For better contrast, the EDX mappings were transformed into grey scale. Bright spots represent chromium-rich grains, black spots refer to pores, and grey refers to the Cr-doped UO_2_ matrix. Scale was the same for all images.

To understand where the Cr is located within the samples, EDS mappings were carried out for the detection of Cr-rich precipitates (Figure 7). A comparison of the EDS mappings and the respective BSE-SEM images indicated no preferred precipitation along the microstructural features (e.g., pores or grain boundaries, cf. example overlay of EDS and BSE image in Figure S3 in supplementary materials). Typically, the ceramics prepared in this study had small Cr-rich precipitates that appeared not to agglomerate to larger particles. The image analyses carried out on the EDS mappings (Table 2) indicated similar precipitate sizes for all samples (i.e., the average sphere equivalent radius calculated from the areas of the precipitates varied between 0.3 and 0.47 µm). The number of Cr-rich precipitates was observed to increase with the doping level, but the total amount of Cr_2_O_3_ in the precipitates remained negligible compared to the total mass doped into the UO_2_ ceramics. The Cr-precipitates in the AREVA pellet were found to be coarser with an average sphere equivalent radius of 0.82 µm. 

Figure 7c shows the Cr-grain distribution of a commercially available Cr doped UO_2_ pellet (AREVA). With an average grain size of 37 µm (Figure 5b, Table S1 in supplementary materials), the microstructure was similar to the pellets produced in the frame of this work doped with 1500 ppm Cr using the wet-coating method and sintered at an oxygen potential of −420 kJ/mol O_2_. 

#### 3.2.2. Cr_2_O_3_-Doping: Structural Uptake and Lattice Parameter Effects

**Figure 8:**
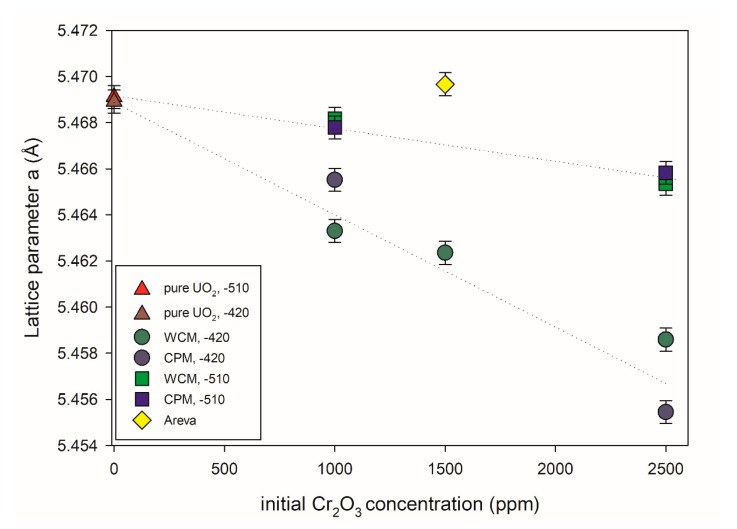
UO_2_ lattice parameters versus initial Cr_2_O_3_ doping level. Two different doping methods and oxygen potentials during the sintering process were investigated. −510: Sinter atmosphere was an Ar/4% H_2_ mixture with an oxygen potential (ΔG° = −RT ln PO_2_) of −510 kJ/mol O_2_. −420: Sinter atmosphere was an Ar/4% H_2_ + Ar/1% H_2_ mixture with an oxygen potential of −420 kJ/mol O_2_. The sintering temperature of all pellets was 1700 °C.

Figure 8 shows the UO_2_ lattice parameter (a) of different pellets synthesized in this work as a function of the initial Cr_2_O_3_ doping level obtained via Rietveld refinement analysis. A Rietveld profile is presented in Figure S4 in supplementary materials for reference. It can be observed that the incorporation of Cr into the UO_2_ lattice led to a subsequent contraction of the lattice parameter, but is dependent on the Cr_2_O_3_-doping level in addition to the oxygen potential used during the sintering process. In contrast, the lattice parameters of pure UO_2_ pellets showed an insignificant change with the variation in the oxygen potential during sintering. In the range of Cr_2_O_3_-doping levels presented here, the increase in the initial Cr_2_O_3_-doping level leads to a nearly linear decrease in the lattice parameters (i.e., a higher amount of Cr_2_O_3_-doping leads to a higher structural uptake of Cr into the lattice of UO_2_). The change in lattice parameters of UO_2_ was less pronounced at lower oxygen potential (−510 kJ/mol O_2_) compared to the samples sintered at higher oxygen potential (−420 kJ/mol O_2_). That general lattice contraction, as observed with increasing initial Cr content, is consistent with previous experimental studies of and Cr doping of UO_2_ [9,21,22,41]. In these references, the lattice contraction was attributed to U(V) formation due to charge balancing of the dopant cations. Accordingly, it can be argued that the greater contraction observed at higher initial Cr content is due to the formation of an increased amount of oxidized U.

The effect of the doping method on the structural uptake appears to play no significant role depending on the oxygen partial pressure. In the case of −510 kJ/mol O_2_, no discernible difference could be observed between the different doping methods used with increasing Cr initial addition. At higher oxygen partial pressure, slight differences could be observed between the lattice parameter when using the different doping methods. At 1000 ppm Cr_2_O_3_ (i.e., below the solubility limit), WCM led to a greater contraction and presumed higher incorporation of Cr, whereas at 2500 ppm Cr_2_O_3_ (i.e., above the solubility limit of Cr), CPM led to greater contraction and presumed higher incorporation of Cr. A mechanistic understanding was beyond the scope of the present investigation, nevertheless, it can be readily observed that the doping methods and controlled oxygen partial pressure greatly assist in incorporation into the UO_2_ matrix as opposed to the sample produced by AREVA via powdering mixing routes where its doping of 1500 ppm Cr_2_O_3_ seemed to have little effect on the UO_2_ lattice and it is argued that the matrix contains little Cr.

## 4. Discussion

**Figure 9:**
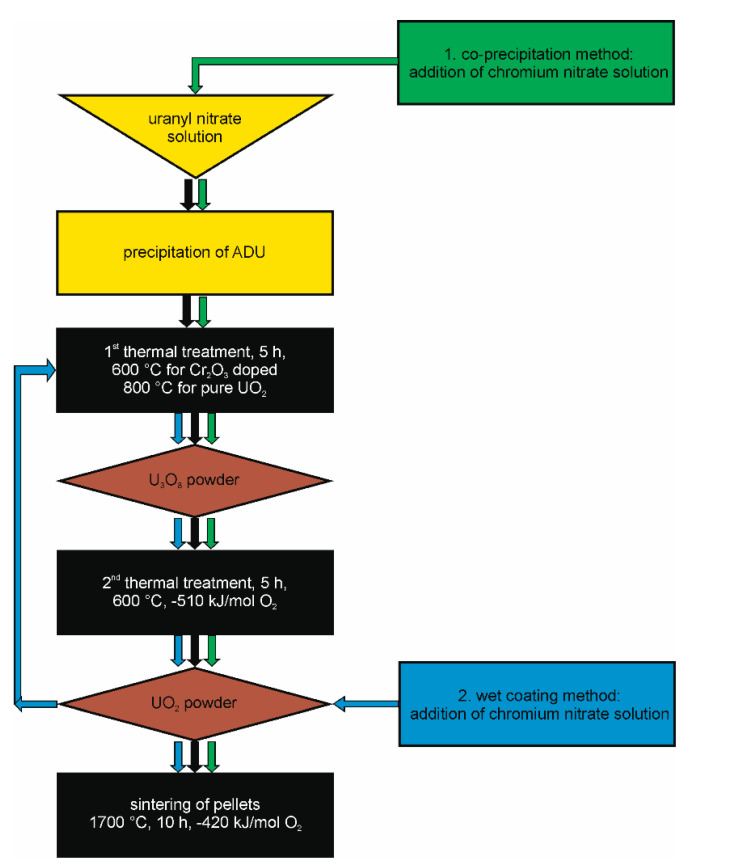
Flow chart of the synthesis route for the production of pure and chromium doped UO_2_ pellets developed in this work.

As already discussed in the introductory part of this paper, the scope of this work is the provision of a “construction kit” for all kinds of UO_2_ model systems useable for single effect studies with the possibility of adjusting parameters such as density/porosity, grainsize, Cr-distribution in the matrix. The flow chart in Figure 9 displays the optimized processing steps for the fabrication of pure UO_2_ and Cr_2_O_3_ doped UO_2_ pellets with well controlled microstructure, sintered density, and distribution of Cr within the pellets. A careful preparation of the three solid phases (powders) ADU, U_3_O_8_, and UO_2_ before the sintering of the ceramic is an essential prerequisite for successful pellet fabrication. In the following section, the parameters that have a strong influence on the sinterability are discussed for the production of pure UO_2_ and of the UO_2_ + Cr_2_O_3_ ceramic via wet chemical methods. In addition, the effects of doping UO_2_ with Cr_2_O_3_ upon the microstructure and crystal structure are discussed in comparison with studies following the powder synthesis route. 

### 4.1. Processing Parameters

The present results for pure UO_2_ indicate that there was a significant effect with respect to the two thermal treatments and the compaction pressure upon sinterability and density of the final ceramic. The optimum compaction pressure for all samples was determined as 637 MPa because it is well below the identified limit of the middle pressure region—independently from the other processing steps [32]. For pure UO_2_, conversion temperatures from ADU to U_3_O_8_ of 600 °C or 800 °C result in a good sinterability. This is well in line with the XRD measurements indicating a full transition to U_3_O_8_ already at 600 °C and the findings of Hung et al. (2001) [27], who also suggested these two temperatures based on their ADU to UO_2_ pellet processing study. 

To minimize the known chromium loss due to volatilization during thermal treatment [9,10], 600 °C was the temperature chosen for the conversion of ADU to U_3_O_8_ when the Cr-doped ceramics were prepared. For the reduction step, a temperature of 600 °C was determined to be sufficient. It is known that higher temperatures during thermal treatment lower the active surface of powders and thereby their reactivity due to the fusion of grains and beginning the sintering processes [42,43]. As no milling or grinding steps should be included in the synthesis route, this effect could change and deteriorate the density/porosity of the sintered pellets [44]. To optimize the sintered density, the sintering atmosphere was optimized in the last step to −420 kJ/mol O_2_. At these conditions, pure UO_2_ pellets with a density of higher than 96 % were prepared, providing the starting point for the optimized Cr-doped pellet route.

Cr-doping was found to increase the sintered density in all cases when compared with the respective UO_2_ reference. For the Cr-doped materials sintered at −510 kJ/mol O_2_, as usually done in other studies [21,22], the relationship between the processing parameters and the final density was more similar to pure UO_2_ in the case of the CPM approach than with the WCM approach. At optimized conditions, the highest densities were reached with the WCM method, independent from the compaction pressure, whereas the CPM method yielded lower sintered densities. The Cr-doped pellets of Silva et al. (2021) [21] reached about 96% of the theoretical density at comparable conditions, close to the pellets prepared via CPM at −510 kJ/mol O_2_. In contrast, the samples prepared by Milena-Perez et al. (2021) [22], which were sintered at an oxygen potential of about −510 kJ/mol O_2_, showed increasing porosity with increasing doping, as observed via SEM.

The increase of the oxygen potential to −420 kJ/mol O_2_ during sintering improved the densities to around 98%, confirming that the combination of 600/600 °C for the thermal treatments with this oxygen potential leads to better results. The densities were similar for WPM and CPM and independent of the doping level. 

### 4.2. Microstructure, Grain Size, Chromium Distribution, and Lattice Parameters in Comparison with the Literature

After the optimization of parameters, the wet chemical synthesis route leads to ceramics with a very well-developed microstructure. All samples, pure and Cr-doped UO_2_, exhibited a uniform grain size distribution and 120° triple points, indicating well equilibrated grain boundaries. Pores were homogeneously distributed with an average size of 1 µm. The mean grain size of the pure UO_2_ was very similar to the samples prepared by Silva et al. (2021) [21] via the classical powder method. Depending on the doping method, during sintering at −510 kJ/mol O_2_, the grain size increased with the doping level of Cr_2_O_3_. In agreement with the observed increasing density, the grain size of the WCM sample was also higher. This was also true for sintering at −420 kJ/mol O_2_, but not as significantly. The values of Milena-Perez et al. (2021) [22] followed a very similar trend as the new data presented here, whereas Bourgeois et al. (2001) [23] produced larger grains in general, which may partly be explained by the advanced spray coating doping method used. Still, the trend of their data was also similar with increasing doping level. The effect of sintering time on the grain growth seems to be insignificant compared to the effect of doping level and oxygen potential, as the sintering time used in [22] and [23] was only 4 h, whereas the sintering time in this work was 10 h. The heating and cooling rates as well as the sinter temperature ([22] 1675 °C [23], and this work 1700 °C) in all studies was in the same order of magnitude. Silva et al. (2021) [21] only saw a very slight change in the mean grain size with increasing Cr-doping. In particular, at higher doping levels above 2000 ppm, their trend in the mean grain size deviated from the trend of Bourgeois [23] and this study. However, the absolute numbers of the mean grain sizes deviated between all studies, indicating that the details of the preparation for the classical powder route also have a significant effect on the resulting microstructure.

Chromium precipitates are a common feature of Cr-doped UO_2_ ceramics. The occurrence of Cr-precipitates and their size was evaluated by Cardinaels et al. (2012) [9] to correlate with the total amount of Cr doping, with a typical size of the precipitates of about 1 to 3 µm, often bound to pores. Similarly, the precipitates in the AREVA reference pellets had Cr-precipitates of about 1 µm in size. For the pellets synthesized in this work, the number and the size of pores was smaller and the number of Cr-precipitates lower. Additionally, the grain size distribution of Cr-grains was more even. The Cr precipitates were typically smaller than 1 µm when produced via wet-chemical routes.

The solubility of chromium in the UO_2_ lattice is known to depend on the oxygen potential [8]. Consequently, the effect of Cr upon the lattice parameter was systematically examined at different Cr initial doping levels for the materials sintered at −420 kJ/mol O_2_ compared to those sintered at −510 kJ/mol O_2_ [9,10,21]. It was argued recently by Riglet-Martial et al. (2014) [8] that the incorporation of Cr into the UO_2_ lattice depends on the oxygen partial pressure whereby higher oxygen potentials lead to higher Cr inclusion; however, volatility of Cr also plays a significant role. That we observed greater lattice contraction and by extension Cr inclusion at higher oxygen potentials is consistent with the argument given by [8]. A full mechanistic understanding was beyond the scope of the present investigation, nevertheless, it can be readily observed that the doping methods and controlled oxygen partial pressure greatly assisted in Cr incorporation into the UO_2_ matrix. In contrast to this observation, the sample produced by AREVA via powder mixing routes with 1500 ppm initial Cr_2_O_3_ doping only seemed to have little effect on the UO_2_ lattice and it is argued that the matrix contained little Cr.

As described earlier, it was argued by Sun et al. that the incorporation of Cr into the UO_2_ matrix resulted in the occurrence of Cr^2+^ with U^5+^ and an oxygen vacancy surrounding it, independent of Cr_2_O_3_ precipitates. It was subsequently argued through this structural and redox mechanism of incorporation that it would result in a relative change of lattice parameter, Δ*a*/*a*, at 1000 ppm of −0.25 − 10 ^−3^ compared to 0 ppm. At the same concentration from the present study, the experimentally determined Δ*a*/*a* derived from Rietveld refinements against XRD data of 0 and 1000 ppm Cr was −3.08 × 10 ^−3^ for WCM −420 kJ/mol O_2_, –0.52 × 10 ^−3^ for WCM −510 kJ/mol O_2_, −1.86 × 10 ^−3^ for CPM −420 kJ/mol O_2_ and −0.72 × 10 ^−3^ for CPM −510 kJ/mol O_2_. Compared to the value reported by [24], the values were somewhat consistent for more reducing conditions, but an order of magnitude difference was observed for oxidizing. However as shown by this investigation, the solubility of Cr is oxygen potential dependent, such that varying the oxygen potential will result in variable amounts of Cr entering the matrix and subsequently effecting the lattice parameter to different degrees. Accordingly, it is difficult to corroborate the proposed structural redox scheme of Cr^2+^ with U^5+^ and an oxygen vacancy surrounding it by [24] via measurement of the lattice parameter due to its dependence on the oxygen potential. Subsequently, it is argued that for true determination of the structural and redox state within the matrix of Cr doped UO_2_, direct measurement of the crystal lattice ideally via grain or a single crystal samples is necessary.

The analysis here indicates that the structural uptake of Cr has no effect on the microstructure and sintered density. This indicates that Cr is surface active during the sintering, changing the behavior of the moving grain boundaries. However, the extent of Cr uptake was found to be strongly dependent upon treatment approach and the oxygen partial pressure.

A comparison of grain boundaries between pure and Cr-doped UO_2_ pellets revealed a distinct curvature of the grain boundaries for the pure UO_2_ pellets, whereas the chromium doped UO_2_ pellets, especially the pellets doped with WCM, had straight grain boundaries. This is an indication for a wetting effect caused by the chromium dopant during sintering [42,43].

## 5. Conclusions

In this work, a wet chemical synthesis route for the production of pure and Cr-doped UO_2_ pellets was demonstrated. Two different approaches for the Cr-doping were followed, namely doping by co-precipitation with the ADU precursor and doping by wet coating of UO_2_ powders. Using an initial synthesis route involving precipitation of ADU as opposed to traditional powder mixing was demonstrated to be advantageous for producing high quality ceramics regarding the material properties and dopant homogeneity to be used later for single effect studies such as dissolution experiments on simulated (spent) nuclear fuel. Such a method can be readily applied to produce high quality ceramics involving other homogeneously distributed dopants relevant to model systems studies such as U-233 or Pu-238, among others.

Comparison with the literature shows similar trends for the influence of Cr-doping on the UO_2_ grain growth in sintered pellets. This effect is pronounced in different degrees depending on the synthesis route. Choosing the wet chemical route of this work allows for a very good reproducibility of the results compared to routes with mechanical steps such as milling.

A recently discussed topic is the structural uptake of Cr into the UO_2_ structure lattice. The theoretical approach of [8,24] describes that chromium incorporation is strongly dependent on oxygen partial pressure during the sintering process. This observation is supported by the findings of this work, although the solubility limit of Cr in the UO_2_ lattice was not determined. The determination of the solubility of chromium in the UO_2_ lattice is an important task for future work.

The precise knowledge of the influence of the synthesis parameters, for instance, the thermal treatment of precursors, method and amount of chromium doping, and oxygen potential during the sintering of the pellets facilitates the production of materials with a hand tailored microstructure with respect to the density, grain size, and chromium grain distribution.

## Figures and Tables

**Table 1:** Properties of Cr_2_O_3_ doped pellets sintered under −420 kJ/mol O_2_.

Name	1st tt	2nd tt	Doping Level	Doping Method	Green Density	Sintered Density	Mean Grain Size
	(°C)		(ppm Cr_2_O_3_)		(%)	(%)	(µm)
1000 ppm Cr_2_O_3_ CPM, 600/600, −420 kJ_1	600	600	1000	CPM	59.5 ± 0.5	97.0 ± 0.5	20 ± 5
2500 ppm Cr_2_O_3_ CPM, 600/600, −420 kJ_1	600	600	2500	CPM	58.0 ± 0.5	98.0 ± 0.5	56 ± 6
1000 ppm Cr_2_O_3_ WCM, 600/600, −420 kJ_1	600	600	1000	WCM	58.9 ± 0.5	98.3 ± 0.5	24 ± 2
1500 ppm Cr_2_O_3_ WCM, 600/600, −420 kJ_4	600	600	1500	WCM	57.0 ± 0.5	98.3 ± 0.5	38 ± 4
2500 ppm Cr_2_O_3_ WCM, 600/600, −420 kJ_1	600	600	2500	WCM	57.8 ± 0.5	98.3 ± 0.5	69 ± 7

1st tt = temperature at first thermal treatment, 2nd tt = temperature at second thermal treatment.

**Table 2:** Image analysis results of the Cr-rich precipitates from the Cr-K-EDS mapping.

		Image Analysis						
Sample	Mass Cr_2_O_3_ Initial	Area Cr_2_O_3_	Number of Particles	Mean Major Axis	Mean Minor Axis	Mean Area	Equ. Radius	Mass Cr_2_O_3_ Precipitate
	(MA%)	(Area%)		(µm)	(µm)	(µm²)	(µm)	(MA%)
1000 ppm Cr_2_O_3_ CP	0.10	0.018	38	0.70	0.49	0.305	0.31	0.0001
1000 ppm Cr_2_O_3_ WC	0.10	0.025	33	0.75	0.60	0.473	0.39	0.0002
AREVA	0.15	0.067	80	1.65	0.71	2.114	0.82	0.0002
1500 ppm Cr_2_O_3_ WC	0.15	0.042	38	0.90	0.79	0.697	0.47	0.0003
2500 ppm Cr_2_O_3_ CP	0.25	0.422	171	0.80	0.56	0.422	0.37	0.0007
2500 ppm Cr_2_O_3_ WC	0.25	0.221	195	0.99	0.76	0.660	0.46	0.0013

## Data Availability

Data is contained within the article and supplementary material.

## Supplementary Materials

The following are available online at https://www.mdpi.com/article/10.3390/ma14206160/s1, Table S1: Values of all experiments; Figure S1: BSE images of a pure UO_2_ pellets sintered at 1700 °C and an oxygen potential of −510 kJ/mol O_2_, for 10 h; Figure S2: BSE images of a Cr-doped UO_2_ pellets with 1500 ppm Cr_2_O_3_ WCM, sintered at 1700 °C and an oxygen potential of −420 kJ/mol O_2_, for 10 h; Figure S3: Example overlay of EDX and BSE image from sample 2500 ppm Cr₂O₃ WCM, 600/600, −420 kJ_1; Figure S4: Example Rietveld profile of UO_2_ doped with Cr at 1000 ppm initial using a WCM approach. Black crosses = observed; red solid line = calculated; red line below = difference curve; vertical black tick marks = positions of the space group allowed Bragg reflections.
