# Supplementary material for: Chromium Doped UO2-Based Ceramics: Synthesis and Characterization of Model Materials for Modern Nuclear Fuels
Source: Materials (Basel). 2021 Oct 17;14(20):6160. doi: 10.3390/ma14206160 (PMC8537854; doi:10.3390/ma14206160)
Supplement: Supplementary file 1 [file materials-14-06160-s001.zip › materials-1374945-supplementary.pdf]

Table S1: Values of all experiments

Name	composition	1st tt (°C)	2nd tt (°C)	comp. pres. (MPa)	doping level (ppm Cr_2O_3)	doping method	oxygen potential during sintering	green density (%)	uncertainty	sintered density (%)	uncertainty	average grain size	uncertainty
pure UO_2, 800/600, -510 kJ_1	pure UO_2	800	600	191.25	pure UO_2	-	-510 kJ	56.7	0.5	91.9	0.5	12 µm	1.2
pure UO_2, 800/600, -510 kJ_2	pure UO_2	800	600	255	pure UO_2	-	-510 kJ	57.7	0.5	92.9	0.5	12 µm	1.2
pure UO_2, 800/600, -510 kJ_3	pure UO_2	800	600	318.75	pure UO_2	-	-510 kJ	59.4	0.5	93.8	0.5	12 µm	1.2
pure UO_2, 800/600, -510 kJ_4	pure UO_2	800	600	382.5	pure UO_2	-	-510 kJ	60.1	0.5	94.8	0.5	12 µm	1.2
pure UO_2, 800/600, -510 kJ_5	pure UO_2	800	600	446.25	pure UO_2	-	-510 kJ	60.7	0.5	95.2	0.5	12 µm	1.2
pure UO_2, 800/600, -510 kJ_6	pure UO_2	800	600	510	pure UO_2	-	-510 kJ	62.1	0.5	95.6	0.5	12 µm	1.2
pure UO_2, 800/600, -510 kJ_7	pure UO_2	800	600	637.5	pure UO_2	-	-510 kJ	63.1	0.5	96.5	0.5	12 µm	1.2
pure UO_2, 800/600, -510 kJ_8	pure UO_2	800	600	765	pure UO_2	-	-510 kJ	64.5	0.5	96.7	0.5	12 µm	1.2
pure UO_2, 800/800, -510 kJ_1	pure UO_2	800	800	191.25	pure UO_2	-	-510 kJ	59.8	0.5	86.5	0.5	12 µm	1.2
pure UO_2, 800/800, -510 kJ_2	pure UO_2	800	800	255	pure UO_2	-	-510 kJ	61.0	0.5	87.9	0.5	12 µm	1.2
pure UO_2, 800/800, -510 kJ_3	pure UO_2	800	800	318.75	pure UO_2	-	-510 kJ	62.8	0.5	89.3	0.5	12 µm	1.2
pure UO_2, 800/800, -510 kJ_4	pure UO_2	800	800	382.5	pure UO_2	-	-510 kJ	64.1	0.5	90.4	0.5	12 µm	1.2
pure UO_2, 800/800, -510 kJ_5	pure UO_2	800	800	446.25	pure UO_2	-	-510 kJ	64.8	0.5	91.0	0.5	12 µm	1.2
pure UO_2, 800/800, -510 kJ_6	pure UO_2	800	800	510	pure UO_2	-	-510 kJ	65.4	0.5	91.7	0.5	12 µm	1.2
pure UO_2, 800/800, -510 kJ_7	pure UO_2	800	800	637.5	pure UO_2	-	-510 kJ	67.4	0.5	92.6	0.5	12 µm	1.2
pure UO_2, 800/800, -510 kJ_8	pure UO_2	800	800	765	pure UO_2	-	-510 kJ	68.7	0.5	93.2	0.5	12 µm	1.2
pure UO_2, 800/900, -510 kJ_1	pure UO_2	800	900	191.25	pure UO_2	-	-510 kJ	60.2	0.5	88.5	0.5	12 µm	1.2
pure UO_2, 800/900, -510 kJ_2	pure UO_2	800	900	255	pure UO_2	-	-510 kJ	61.7	0.5	90.1	0.5	12 µm	1.2
pure UO_2, 800/900, -510 kJ_3	pure UO_2	800	900	318.75	pure UO_2	-	-510 kJ	62.8	0.5	91.1	0.5	12 µm	1.2
pure UO_2, 800/900, -510 kJ_4	pure UO_2	800	900	382.5	pure UO_2	-	-510 kJ	64.1	0.5	91.7	0.5	12 µm	1.2
pure UO_2, 800/900, -510 kJ_5	pure UO_2	800	900	446.25	pure UO_2	-	-510 kJ	64.7	0.5	92.5	0.5	12 µm	1.2
pure UO_2, 800/900, -510 kJ_6	pure UO_2	800	900	510	pure UO_2	-	-510 kJ	65.9	0.5	92.8	0.5	12 µm	1.2
pure UO_2, 800/900, -510 kJ_7	pure UO_2	800	900	637.5	pure UO_2	-	-510 kJ	67.3	0.5	93.5	0.5	12 µm	1.2
pure UO_2, 800/900, -510 kJ_8	pure UO_2	800	900	765	pure UO_2	-	-510 kJ	67.9	0.5	94.0	0.5	12 µm	1.2
pure UO_2, 600/600, -510 kJ_1	pure UO_2	600	600	191.25	pure UO_2	-	-510 kJ	50.6	0.5	89.3	0.5	12 µm	1.2
pure UO_2, 600/600, -510 kJ_2	pure UO_2	600	600	255	pure UO_2	-	-510 kJ	53.3	0.5	90.9	0.5	12 µm	1.2

pure UO_2, 600/600, -510 kJ_3	pure UO_2	600	600	318.75	pure UO_2	-	-510 kJ	54.6	0.5	91.9	0.5	12 µm	1.2
pure UO_2, 600/600, -510 kJ_4	pure UO_2	600	600	382.5	pure UO_2	-	-510 kJ	56.1	0.5	92.7	0.5	12 µm	1.2
pure UO_2, 600/600, -510 kJ_5	pure UO_2	600	600	446.25	pure UO_2	-	-510 kJ	57.2	0.5	93.5	0.5	12 µm	1.2
pure UO_2, 600/600, -510 kJ_6	pure UO_2	600	600	510	pure UO_2	-	-510 kJ	58.0	0.5	93.8	0.5	12 µm	1.2
pure UO_2, 600/600, -510 kJ_7	pure UO_2	600	600	637.5	pure UO_2	-	-510 kJ	59.8	0.5	94.5	0.5	12 µm	1.2
pure UO_2, 600/600, -510 kJ_8	pure UO_2	600	600	765	pure UO_2	-	-510 kJ	60.8	0.5	94.6	0.5	12 µm	1.2
pure UO_2, 600/600, -420 kJ_1	pure UO_2	600	600	637.5	pure UO_2	-	-420 kJ	61.3	0.5	96.3	0.5	12 µm	1.2
pure UO_2, 600/600, -420 kJ_2	pure UO_2	600	600	637.5	pure UO_2	-	-420 kJ	61.9	0.5	96.3	0.5	12 µm	1.2
1000 ppm Cr_2O_3 CPM, 600/600, -510 kJ_1	Cr doped UO_2	600	600	191.25	1000 ppm Cr_2O_3	CPM	-510 kJ	52.8	0.5	94.1	0.5	12 µm	2
1000 ppm Cr_2O_3 CPM, 600/600, -510 kJ_2	Cr doped UO_2	600	600	318.75	1000 ppm Cr_2O_3	CPM	-510 kJ	56.0	0.5	95.6	0.5	12 µm	2
1000 ppm Cr_2O_3 CPM, 600/600, -510 kJ_3	Cr doped UO_2	600	600	446.25	1000 ppm Cr_2O_3	CPM	-510 kJ	57.9	0.5	96.6	0.5	12 µm	2
1000 ppm Cr_2O_3 CPM, 600/600, -510 kJ_4	Cr doped UO_2	600	600	510	1000 ppm Cr_2O_3	CPM	-510 kJ	59.4	0.5	96.9	0.5	12 µm	2
1000 ppm Cr_2O_3 CPM, 600/600, -510 kJ_5	Cr doped UO_2	600	600	637.5	1000 ppm Cr_2O_3	CPM	-510 kJ	60.9	0.5	97.3	0.5	12 µm	2
1000 ppm Cr_2O_3 CPM, 600/600, -510 kJ_6	Cr doped UO_2	600	600	765	1000 ppm Cr_2O_3	CPM	-510 kJ	61.8	0.5	97.6	0.5	12 µm	2
2500 ppm Cr_2O_3 CPM, 600/600, -510 kJ_1	Cr doped UO_2	600	600	637.5	2500 ppm Cr_2O_3	CPM	-510 kJ					27.4 µm	5.6
2500 ppm Cr_2O_3 CPM, 600/600, -510 kJ_2	Cr doped UO_2	600	600	637.5	2500 ppm Cr_2O_3	CPM	-510 kJ					27.4 µm	
2500 ppm Cr_2O_3 CPM, 600/600, -510 kJ_3	Cr doped UO_2	600	600	637.5	2500 ppm Cr_2O_3	CPM	-510 kJ					27.4 µm	
1000 ppm Cr_2O_3 WCM, 600/600, -510 kJ_1	Cr doped UO_2	600	600	191.25	1000 ppm Cr_2O_3	WCM	-510 kJ	51.4	0.5	97.9	0.5	19 µm	2
1000 ppm Cr_2O_3 WCM, 600/600, -510 kJ_2	Cr doped UO_2	600	600	318.75	1000 ppm Cr_2O_3	WCM	-510 kJ	54.6	0.5	98.3	0.5	19 µm	2
1000 ppm Cr_2O_3 WCM, 600/600, -510 kJ_3	Cr doped UO_2	600	600	382.5	1000 ppm Cr_2O_3	WCM	-510 kJ	55.6	0.5	98.4	0.5	19 µm	2
1000 ppm Cr_2O_3 WCM, 600/600, -510 kJ_4	Cr doped UO_2	600	600	446.25	1000 ppm Cr_2O_3	WCM	-510 kJ	57.3	0.5	98.3	0.5	19 µm	2
1000 ppm Cr_2O_3 WCM, 600/600, -510 kJ_5	Cr doped UO_2	600	600	510	1000 ppm Cr_2O_3	WCM	-510 kJ	57.9	0.5	98.4	0.5	19 µm	2
1000 ppm Cr_2O_3 WCM, 600/600, -510 kJ_6	Cr doped UO_2	600	600	573.75	1000 ppm Cr_2O_3	WCM	-510 kJ	59.1	0.5	98.5	0.5	19 µm	2
1000 ppm Cr_2O_3 WCM, 600/600, -510 kJ_7	Cr doped UO_2	600	600	637.5	1000 ppm Cr_2O_3	WCM	-510 kJ	60.0	0.5	98.6	0.5	19 µm	2
1000 ppm Cr_2O_3 WCM, 600/600, -510 kJ_8	Cr doped UO_2	600	600	765	1000 ppm Cr_2O_3	WCM	-510 kJ	61.5	0.5	98.6	0.5	19 µm	2
2500 ppm Cr_2O_3 WCM, 600/600, -510 kJ_1	Cr doped UO_2	600	600	637.5	2500 ppm Cr_2O_3	WCM	-510 kJ					45.8 µm	4.5
2500 ppm Cr_2O_3 WCM, 600/600, -510 kJ_2	Cr doped UO_2	600	600	637.5	2500 ppm Cr_2O_3	WCM	-510 kJ					45.8 µm	4.5
2500 ppm Cr_2O_3 WCM, 600/600, -510 kJ_3	Cr doped UO_2	600	600	637.5	2500 ppm Cr_2O_3	WCM	-510 kJ					45.8 µm	4.5
1000 ppm Cr_2O_3 CPM, 600/600, -420 kJ_1	Cr doped UO_2	600	600	637.5	1000 ppm Cr_2O_3	CPM	-420 kJ	59.5	0.5	96.9	0.5	20	5
1000 ppm Cr_2O_3 CPM, 600/600, -420 kJ_2	Cr doped UO_2	600	600	637.5	1000 ppm Cr_2O_3	CPM	-420 kJ	59.4	0.5	97.0	0.5	20	5

2500 ppm Cr_2O_3 CPM, 600/600, -420 kJ_1	Cr doped UO_2	600	600	637.5	2500 ppm Cr_2O_3	CPM	-420 kJ	58.0	0.5	98.1	0.5	56	5.6
2500 ppm Cr_2O_3 CPM, 600/600, -420 kJ_2	Cr doped UO_2	600	600	637.5	2500 ppm Cr_2O_3	CPM	-420 kJ	58.1	0.5	97.9	0.5	56	5.6
1000 ppm Cr_2O_3 WCM, 600/600, -420 kJ_1	Cr doped UO_2	600	600	637.5	1000 ppm Cr_2O_3	WCM	-420 kJ	58.9	0.5	98.3	0.5	24	2.4
1000 ppm Cr_2O_3 WCM, 600/600, -420 kJ_2	Cr doped UO_2	600	600	637.5	1000 ppm Cr_2O_3	WCM	-420 kJ	58.9	0.5	98.3	0.5	24	2.4
1500 ppm Cr_2O_3 WCM, 600/600, -420 kJ_1	Cr doped UO_2	600	600	573.75	1500 ppm Cr_2O_3	WCM	-420 kJ	56.6	0.5	98.4	0.5	37.81	3.8
1500 ppm Cr_2O_3 WCM, 600/600, -420 kJ_2	Cr doped UO_2	600	600	573.75	1500 ppm Cr_2O_3	WCM	-420 kJ	56.9	0.5	98.5	0.5	37.81	3.8
1500 ppm Cr_2O_3 WCM, 600/600, -420 kJ_3	Cr doped UO_2	600	600	573.75	1500 ppm Cr_2O_3	WCM	-420 kJ	57.3	0.5	98.1	0.5	37.81	3.8
1500 ppm Cr_2O_3 WCM, 600/600, -420 kJ_4	Cr doped UO_2	600	600	573.75	1500 ppm Cr_2O_3	WCM	-420 kJ	56.9	0.5	98.2	0.5	37.81	3.8
1500 ppm Cr_2O_3 WCM, 600/600, -420 kJ_5	Cr doped UO_2	600	600	573.75	1500 ppm Cr_2O_3	WCM	-420 kJ	57.1	0.5	98.4	0.5	37.81	3.8
2500 ppm Cr_2O_3 WCM, 600/600, -420kJ_1	Cr doped UO_2	600	600	637.5	2500 ppm Cr_2O_3	WCM	-420 kJ	57.8	0.5	98.2	0.5	69	6.9
2500 ppm Cr_2O_3 WCM, 600/600, -420 kJ_2	Cr doped UO_2	600	600	637.5	2500 ppm Cr_2O_3	WCM	-420 kJ	57.9	0.5	98.3	0.5	69	6.9
AREVA	Cr doped UO_2	?	?	?	1500 ppm	?	?	?	?	96.2	0.1	37	4.0

1st tt = temperature at first step of thermal treatment, 2nd tt = temperature at second step of thermal treatment, comp. press. = compaction pressure. Uncertainties for the temperature of 1st and 2nd step of thermal treatment is ± 5° C. Uncertainty of the pressure during compaction is 0.1 MPa. Uncertainties of all determined densities are 2 sigma deviations.

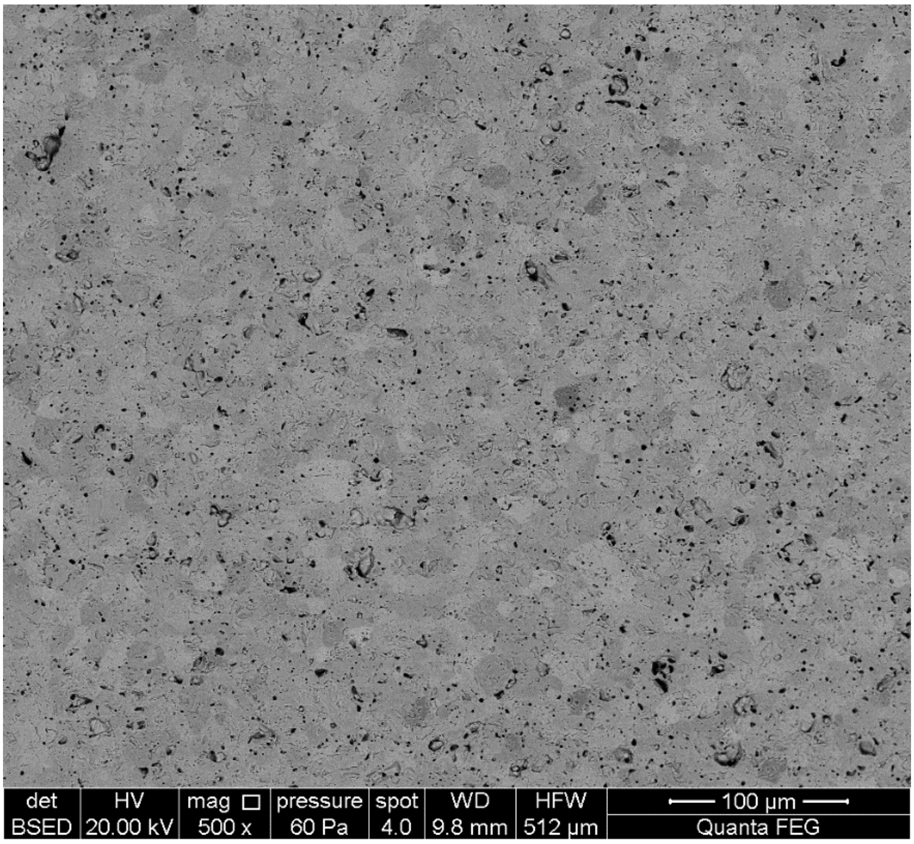

Figure S1: BSE images of a pure UO_2 pellets sintered at 1700 °C and an oxygen potential of -510kJ/mol O_2, for 10 h.

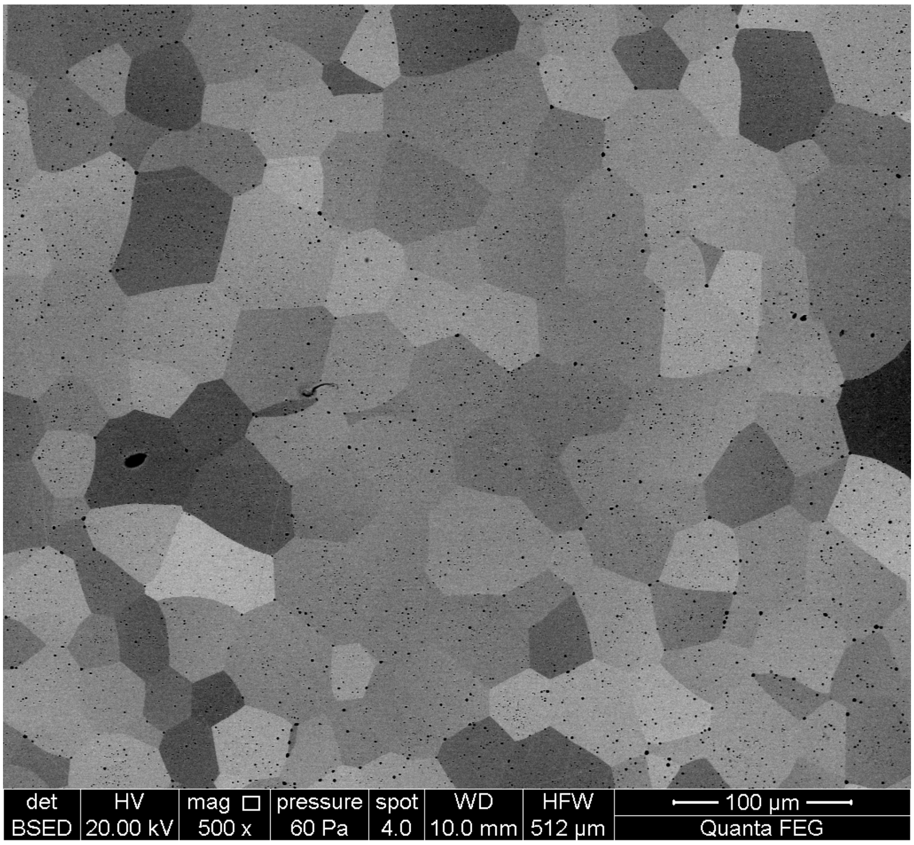

Figure S2: BSE images of a Cr-doped UO_2 pellets with 1500 ppm Cr_2O_3 WCM, sintered at 1700 °C and an oxygen potential of -420kJ/mol O_2, for 10 h.

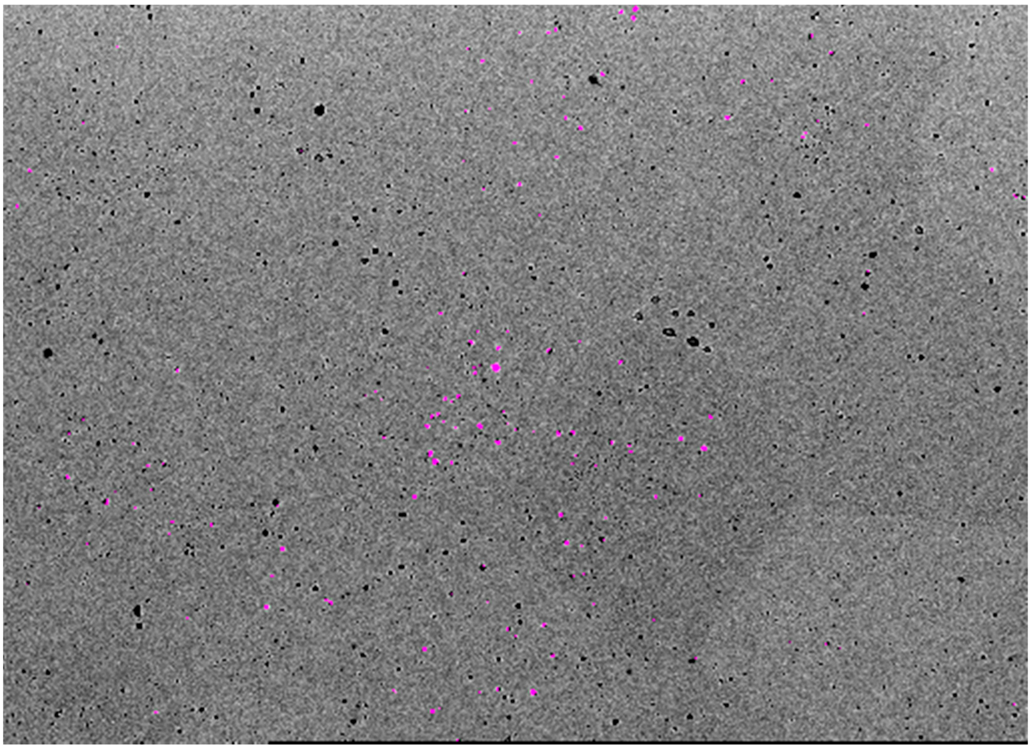

Figure S3: Example overlay of EDX and BSE image from sample 2500 ppm Cr_2O_3 WCM, 600/600, -420kJ_1.

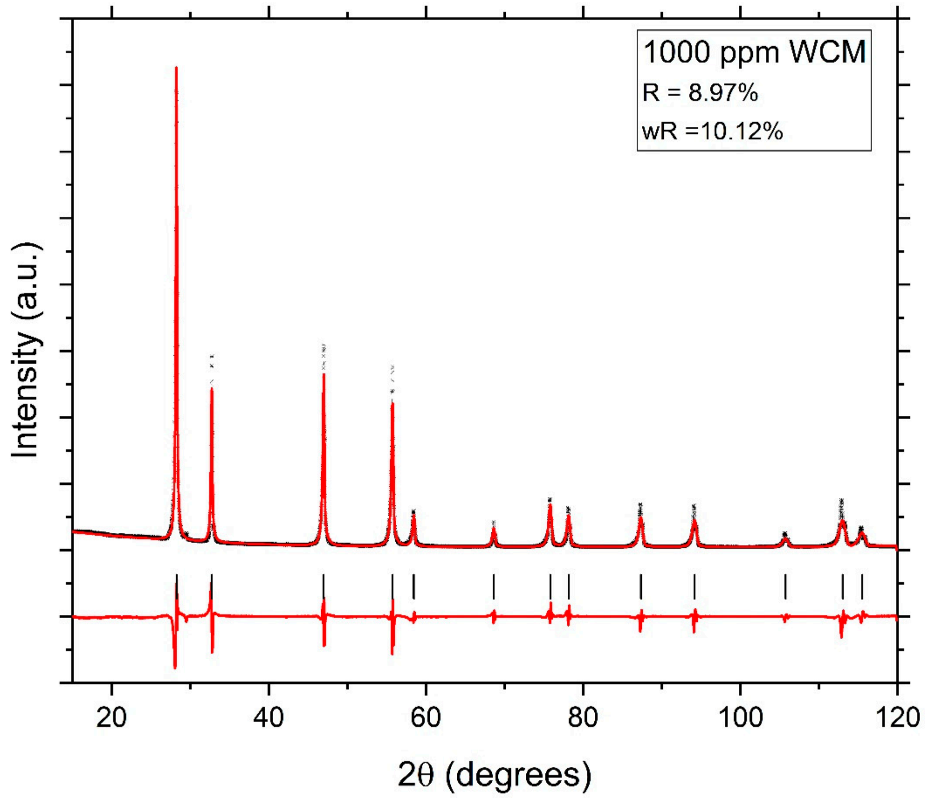

Figure S4: Example Rietveld profile of UO_2 doped with Cr at 1000 ppm initial using a WCM approach. Black crosses = observed; red solid line = calculated; red line below = difference curve; vertical black tick marks = positions of the space group allowed Bragg reflections.
